# Female differential allocation in response to extrapair offspring and social mate attractiveness

**DOI:** 10.1002/ece3.7560

**Published:** 2021-05-25

**Authors:** Kerianne M. Wilson, Nancy Tyler Burley

**Affiliations:** ^1^ Department of Ecology and Evolutionary Biology University of California Irvine CA USA; ^2^ Evolution, Ecology and Organismal Biology Department University of California Riverside CA USA

**Keywords:** differential allocation, extra‐pair offspring, female extra‐pair reproductive success, male quality, parental investment, zebra finch

## Abstract

Renewed debate over what benefits females might gain from producing extra‐pair offspring emphasizes the possibility that apparent differences in quality between within‐pair and extra‐pair offspring are confounded by greater maternal investment in extra‐pair offspring. Moreover, the attractiveness of a female's social mate can also influence contributions of both partners to a reproductive attempt. Here, we explore the complexities involved in parental investment decisions in response to extra‐pair offspring and mate attractiveness with a focus on the female point of view. Adult zebra finches paired and reproduced in a colony setting. A male's early‐life diet quality and his extra‐pair reproductive success were used as metrics of his mating attractiveness. Females paired with males that achieved extra‐pair success laid heavier eggs than other females and spent less time attending their nests than their mates or other females. Extra‐pair nestlings were fed more protein‐rich hen's egg than within‐pair nestlings. Females producing extra‐pair offspring had more surviving sons than females producing only within‐pair offspring. Collectively, results show that females differentially allocate resources in response to offspring extra‐pair status and their social mate's attractiveness. Females may also obtain fitness benefits through the production of extra‐pair offspring.

## Graphical abstract

To understand how female birds benefit from mating with males other than their social partners (extra‐pair mating), we assessed how the production of extra‐pair offspring and the relative attractiveness of their social partners influence maternal caregiving and reproduction. Collectively, results show that females differentially allocate resources in response to offspring extra‐pair status and their social mate's attractiveness. Females may also obtain direct fitness benefits through the production of extra‐pair offspring.

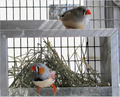

## 1 INTRODUCTION

While the benefits of extra‐pair paternity to males of pair‐bonding species are broadly acknowledged, there is less agreement on possible benefits to females from extra‐pair mating (Forstmeier et al., 2014; Griffith et al., 2002; Kempenaers & Schlicht, 2010). One hypothesis is that females can benefit by producing extra‐pair offspring that are of higher genetic quality (having “good genes”) compared to offspring produced from within‐pair matings (Neff & Pitcher, 2005). While studies on birds have generated some support for this hypothesis (Forstmeier et al., 2002; Fossøy et al., 2008; Hasselquist et al., 1996; Houtman, 1992; Kawano et al., 2008), meta‐analyses and modeling suggest that genetic benefits may be small or absent (Akçay & Roughgarden, 2007; Arnqvist & Kirkpatrick, 2005; Westneat & Stewart, 2003), and the potential for maternal effects (e.g., hatch order, egg hormone level differences) to confound any genetic effects must be considered (Griffith et al., 2002; Jennions & Petrie, 2000; Kempenaers & Schlicht, 2010; Schmoll, 2011).

A likely source of confounding maternal effects is the differential allocation of resources to extra‐pair offspring (EPOs) or clutches containing EPOs versus within‐pair offspring (WPOs) or clutches containing only WPOs (Ferree et al., 2010; Magrath et al., 2009; Schmoll, 2011; Tschirren et al., 2012). Specifically, in species with biparental care, the differential allocation hypothesis predicts that a female will provide higher parental investment to offspring of an attractive or high‐quality male—that is, one that she judges to possess “good genes”—than to an unattractive/low‐quality male (Burley, 1988, 2019; Horvathova et al., 2012; Sheldon, 2000). This hypothesis, typically framed in the context of within‐pair matings, is applicable to investment in EPOs if females seek extra‐pair matings with males that are more attractive than their social mates, as some studies have found (Forstmeier et al., 2002; Kempenaers & Schlicht, 2010; Wilson et al., 2019). The differential allocation hypothesis can be extended to EPO investment because offspring of extra‐pair partners are expected to possess superior heritable traits, including sons that display greater secondary sexual trait expression. An alternative possibility to the “good genes” hypothesis is that greater expression of secondary sexual traits of adult extra‐pair sons results from differential allocation of resources toward these young by mothers (Tschirren et al., 2012). Of course, these possibilities are not mutually exclusive, particularly since a pattern of greater maternal investment in extra‐pair offspring would be paradoxical unless such offspring tend to have higher reproductive value than within‐pair offspring.

Several variables likely interact to influence the extent to which females benefit from extra‐pair matings. To date, studies of maternal investment in response to extra‐pair offspring have most often focused on primary reproductive allocation, notably egg size (Bolund et al., 2009; Krist et al., 2005; Tschirren et al., 2012), which may be positively associated with hatchling survival and development of superior adult trait expression (Krist, 2011; Wagner & Williams, 2007). However, less is known about parental expenditure in extra‐pair offspring during the incubation and provisioning phases. Since social parents often contribute to incubation and offspring provisioning—which can be costly to caregivers (Alonso‐Alvarez et al., 2004; Monaghan & Nager, 1997; Nord & Williams, 2015; Owens & Bennett, 1994; Williams, 2018)—these reproductive phases likely contribute additional sources of variation in allocation of resources to WPOs versus EPOs. A bird's own extra‐pair mating tendencies may also affect its parental investment patterns. Males that sire more EPOs may invest less in their social mates’ clutches due to the time and resources required to seek extra‐pair mates (Ball et al., 2017; Crouch & Mason‐Gamer, 2018) while, as noted above, females may increase investment in broods that contain EPOs (Schmoll, 2011). In addition to the considerations enumerated above, the extent to which preferred male traits are heritable is usually unknown, and environmental conditions typically influence expression of such traits (Cornwallis & Uller, 2010; Griffith et al., 1999).

In this paper, we report results from a breeding experiment in which male breeders had been raised on high‐ or low‐quality diets, with female breeders raised on an intermediate diet. Results from this breeding population focusing on male reproductive performance have been reported elsewhere (Wilson et al., 2019) and show that developmental stress negatively impacts secondary sexual traits and son production. To examine the female perspective here, we include two measures of the quality/attractiveness of females’ social mates. The first is male early diet quality, which positively impacts adult male expression of secondary sexual traits (Naguib & Nemitz, 2007; Wilson et al., 2019); females from this population (Burley et al., 2018) and another (Spencer et al., 2005) prefer males raised under higher‐diet conditions. The second metric is male extra‐pair mating success, which is presumed to be greater for higher‐quality males and has been previously used as an index of attractiveness in studies on this species (Bolund et al., 2009; Houtman, 1992). Male extra‐pair success is thought to reflect genetic as well as environmental determinants of variation (Jennions & Petrie, 2000; Neff & Pitcher, 2005). Since a female's mate choice and reproductive allocation decisions are likely to depend on her mate's developmental history as well as his genetic quality—and she may well lack information to assess the relative contribution of environmental versus heritable effects to male phenotype—use of these two measures may provide insight into a broad range of male phenotypic attributes that influence female extra‐pair mating decisions.

By manipulating early‐life diet quality of male zebra finches and measuring subsequent reproductive investment as well as within‐pair and extra‐pair reproductive success, we address the following questions: Do females invest more in EPOs or EPO‐containing clutches? Do they invest more in WPOs sired by attractive males? Do males invest differentially in offspring care based on their own attractiveness and/or the presence of EPOs in the clutches they raise? In line with the differential allocation hypothesis, we predicted that females with attractive social mates would invest more in offspring at all phases of reproduction and that attractive males would show lower parental care. In light of previous findings for this species (Tschirren et al., 2012), we also expected that females would invest more in EPOs regardless of the quality of their social mates. Lacking evidence that male zebra finches contribute to the EPOs they sire or that they can detect their mates’ EPOs in the clutches they raise, we did not make specific predictions about male caregiving behavior in relation to EPOs. Finally, we ask whether females gained direct fitness benefits through production of EPOs, a possibility that has seldom been addressed empirically, by assessing female overall reproductive success in relation to her extra‐pair success.

## 2 METHODS

### 2.1 Founder rearing conditions and flight initiation

**FIGURE 1:**
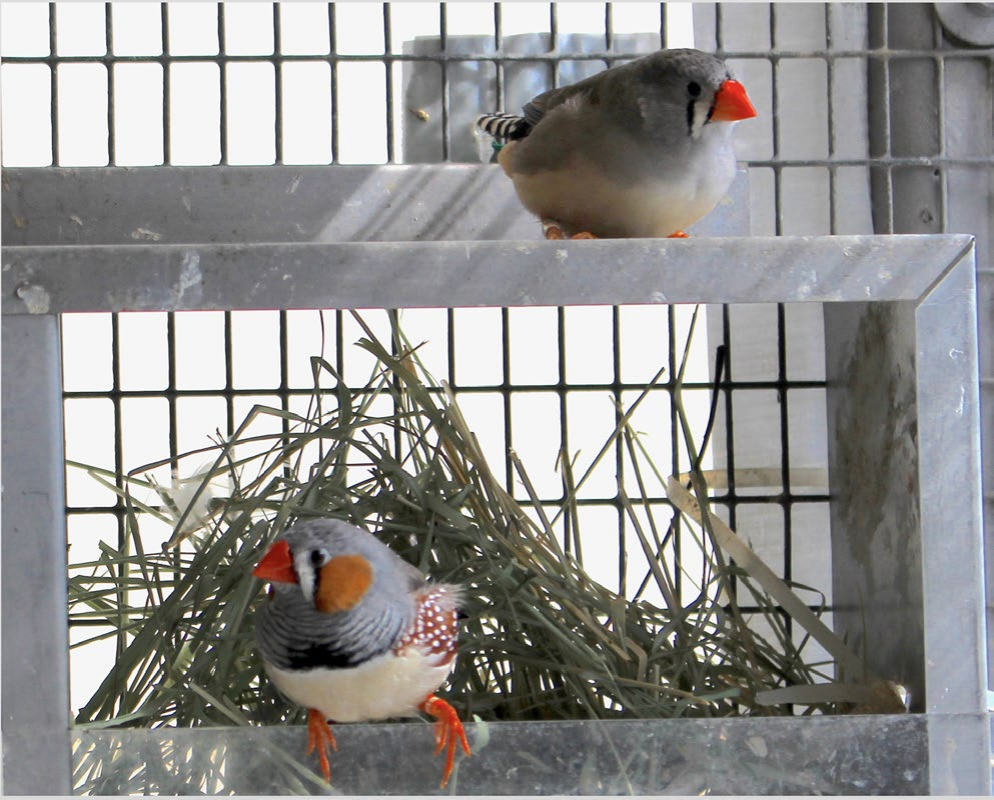
Female and male zebra finches

Zebra finches (Figure 1) selected as founders of this breeding experiment were raised in one of four large outdoor aviary flights (3 × 12 × 2.3 m), each of which contained 64 breeders (32 of each sex). To reduce relatedness between male and female founders and to control founder rearing diet, two of the rearing flights were used to generate female founders, while the remaining flights were used to generate male founders. Access to boiled hen's egg differed among rearing flights, but in other respects (size, resources, microclimate), all rearing flights were virtually identical. Female founders were reared in flights in which hen's egg was made available three times a week (LAB diet). In order to manipulate male early diet quality, male founders were reared in either a flight in which egg was provided daily (HI diet) or one in which egg was never provided (LO diet). All diet regimes were maintained until birds were selected for this study. Hen's egg has an amino acid profile similar to that of half‐ripe grass seed (Allen & Hume, 1997), which is seasonally available to zebra finches in the wild and has a higher protein content than ripe grass seed. All rearing flights provided breeders and fledged offspring with ad libitum access to a commercial mix of ripe grass seed for estrildines, cuttlefish bone, ground oyster shell and water, and green vegetables three times a week. All resources were provided to this ground‐feeding species on the aviary floor.

Offspring produced in rearing flights were banded with numbered, closed metal leg bands when they were 7–14 days old. Once offspring reached 45 +/‐ 3 days of age, they were housed in single‐sex cages (68 × 50 × 54 cm) at standard densities (10 birds) inside their rearing flight until they reached adulthood (100 days of age) in order to provide developing birds visual and acoustic contact with adults; such exposure is important for imprinting on visual and acoustic traits (Bischof et al., 2002; Bolhuis, 1991; Immelmann, 1975). Birds remained in single‐sex cages until they were selected for use in the present study. Additional details on founder rearing conditions and the history of this experimental colony can be found elsewhere (Wilson et al., 2019).

In total, 32 females and 32 (16 HI and 16 LO) males were selected to found the breeding colony for the current experiment. Once selected, birds were held in single‐sex flights and maintained on the LAB diet for 6 weeks before they were released into the breeding flight, a protocol that allowed LO‐diet males the opportunity to become familiar with the availability of hen's egg as a food resource. LO‐ and HI‐diet males did not differ in their consumption of egg during this period (two‐sample *t* test: t = 1.24, *df* = 30 *p* = .22). During this time, male phenotype data were collected and a 25‐μl blood sample was collected from the brachial vein of each bird for genetic parentage analyses. HI‐diet males had a greater expression of two secondary sexual traits compared to those raised on the LO‐diet (redder beaks, larger cheek patches), supporting the use of natal diet as a metric of attractiveness (see Wilson et al., 2019 for details). Founders varied from 6 to 13 months of age (X̄ +/‐ *SD*: females—279 +/‐ 53 days old; males—354 +/‐ 50 days old) at the start of breeding and had no prior breeding experience. All birds were judged to be in excellent overall condition and no more than 2 siblings from each family were employed (males were derived from 20 families and females from 24 families). Lastly, all birds were banded for identification during observations using colors for which zebra finches show no band preference (Burley, 1985).

### 2.2 Breeding conditions and reproductive measures

Founders of both sexes were released simultaneously into a single (3 × 12 × 2.3 m) outdoor aviary flight and allowed to pair and breed for 5 months. The flight was maintained on the LAB diet. Ample nest sites (~2.5/ breeding pair) and nest material (dried Bermuda grass and feathers) were provided. The time it took each pair to establish their first clutch did not vary based on male natal diet or extra‐pair success of either partner (3‐way ANOVA: *F*_(3,21)_ = 0.22; *p* =.87; Table 1). All nest sites were checked daily: New eggs were uniquely marked using a fine tipped Sharpie® and weighed on an electronic balance sensitive to 0.01 gram. Occasionally fresh eggs were not found on the day they were laid, as evidenced by the discovery of two or more unmarked eggs in a nest on the same day; such eggs were not included in analyses of lay order due to ambiguity. Since relatively few eggs in a single clutch hatched on the same day (12.2%), we were able to reliably track the egg from which each offspring hatched by noting which egg was “missing” when a new, unmarked nestling was found. The down feathers of new hatchlings were colored with nontoxic markers to track hatch order. When two nestlings appeared on the same day, the older nestling (assessed by having drier down feathers or, secondarily, weighing more) was assigned to the egg of earlier lay order. Nestlings were banded with seamless numbered bands between 7 and 14 days of age. When independent offspring reached 45 +/‐ 3 days of age, they were caught and housed within the natal flight in cages containing other same‐sex offspring of similar age. At this time, a 25‐μl blood sample was collected from a brachial vein for genetic parentage assignment.

**TABLE 1:** Latency to establish first clutch

Variable	Degrees of Freedom	Sum of Squares (Mean Square of the Error)	*F*‐Value	*p*
Clutch Latency	3, 23		0.23	.87
Clutch Status (EP or WP only)	1	813.79	0.62	.44
Male Extra‐Pair Success	1	21.93	0.02	.90
Male Natal Diet	1	98.44	0.08	.79
Residual	20	26,164.98 (1,308.25)		

3‐way ANOVA (initial model included 3 main effects only). *N* = 24.

### 2.3 Social and genetic parentage assignment

Both genetic parentage and social parentage were tracked in order to identify which birds were successful in producing surviving EPOs. Social parentage was assigned to each clutch through regular observations of active nests beginning when eggs first appeared in a nest and ending once the last nestling fledged. Genetic analyses involved isolation and amplification of DNA through PCR with fluorescently labeled primers corresponding to 8 highly polymorphic microsatellite loci previously identified for zebra finches (Forstmeier et al., 2007). CERVUS 3.0 (Kalinowski et al., 2007) was used to assign parentage based on correspondence at these 8 loci. Genetic analyses of blood samples collected from all founders and 186 offspring were used to assign genetic parentage based on 6 or more unambiguous loci.

Because only offspring that survived to independence were genotyped, clutches categorized here as containing only WPOs may have contained EPOs that died before independence. The consequence of such misclassification is that results are conservative for predicted patterns of parental investment. To further minimize ambiguity, all analyses of parental investment focus on offspring that survived to independence and the clutches from which all surviving offspring were genotyped. This criterion excluded clutches containing surviving offspring that were not genotyped and resulted in the inclusion of 55 clutches from 25 pairs (during the study period, 76 clutches from 28 pairs produced at least one surviving offspring). Of the male breeders included, 12 were raised on the HI diet and 13 on the LO diet. Nine females and 9 males (3 LO and 6 HI) raised one or more EPOs to independence during the course of the experiment (range: 1–6 EPOs).

### 2.4 Parental nest attendance

The amount of time parents spent attending their nests was recorded for a subset of nests (*N* = 28) and pairs (*N* = 18) to quantify parental allocation during the incubation phase. Nests containing at least one egg were selected for parental attendance observations if no egg had yet hatched. Observers recorded data from inside the aviary and were careful to minimize disturbance of incubating birds. Observers conducted 30‐min, all‐accounts samples of the amount of time each parent spent inside its nest. Thus, “nest attendance time” included time devoted to incubation, nest construction and possibly nest defense. Nests included in analyses were observed on 2 to 6 different days (X̄ +/‐ *SD*: 5.32 +/‐ 1.02) during the incubation phase (observation day: X̄ +/‐ *SD*: 3.07 + /‐3.89, with day 0 being the day the last egg was laid; the first egg hatched on about day 11) and contained one or more offspring that survived to independence. Nest observations were discontinued after the first egg hatched.

### 2.5 Nestling provisioning

Nestling provisioning was recorded in order to examine whether it differed based on nestling status (EP or WP) and/or male attractiveness (male natal diet and extra‐pair success). Since seed and egg differ in nutrient quality, we measured provisioning of these food types separately on days when egg was provided, so that relative amounts of egg versus seed feeding could be assessed. Immediately prior to provisioning sampling sessions, founders were provided with a bowl of egg placed on the aviary floor and allowed to feed for 3 min. If birds flushed in response to a disturbance, timing was suspended until bird(s) returned to the egg bowl. The bowl was then removed and, after an additional 5 min, experimenters entered the aviary and scored crop contents of all nestlings. This protocol was repeated to produce 2 samples of nestling provisioning on each sampling day. At the end of the testing period, the remaining egg was left in the flight for the rest of the day. This design was selected after preliminary trials confirmed that (1) increasing the foraging interval from 3 to 5 min did not significantly increase the number of adults that consumed egg (although the same individuals often revisited food bowls more times) and (2) interference competition at the food bowl was uncommon (see Discussion).

Nestlings ranged in age from 0 (hatching) to 15 days (X̄ +/‐ *SD*: 6.28 +/‐ 3.53 days of age, *N* = 44 nestlings); such nestlings have translucent skin through which contents can be unambiguously scored. The amounts of egg and seed present in the crop were each assessed on a 7‐point scale ranging from 0 (“empty crop”) to 3 (“full crop”), with 0.5‐point increments. Crop volume increases as nestlings grow, so these values represent the proportion of the crop filled with a given food type. Nestling provisioning scores were averaged across the two daily samples. Nestlings present during sampling were scored, on average, on 2.09 +/‐ 1.12 days.

### 2.6 Analyses

Repeated‐measures, linear mixed‐effect models (RM LMMs) were used to investigate the impact of the three main effects under consideration (male diet, male extra‐pair success and female extra‐pair success) on parental investment at the egg and nestling stages: egg mass, clutch size, nest attendance time, and nestling provisioning of seed and egg. While the two measures of male attractiveness (extra‐pair success and diet history) may be expected to covary, they were only weakly related here (Fisher's exact: *p* = .057).

In these models, which are described in more detail below, the measure of “male extra‐pair success” is a dichotomous variable: either he sired one or more surviving EPOs to independence (successful) or he did not (unsuccessful). The measure of female extra‐pair offspring success varied among models depending on the dependent variable. Unless otherwise indicated, analyses of interactions were limited to only *a priori* expectations of diet effects on measures of reproductive investment to reduce the potential type I error. Nonsignificant interaction effects and covariates (α > 0.05) were removed from models in a reverse stepwise process beginning with interactions. No fixed effect or random effect was removed from any analysis. Variance inflation factors were further assessed for all analyses to ensure limited effects of multicollinearity (all VIFs <1.36).

The RM LMM predicting egg mass included the three main effects, with female extra‐pair success included as the fixed effect “Egg Status (EP or WP)”; egg status was assigned to each individual egg based on offspring genetic analysis. Lay order was included as a covariate, and mother's identity was included as a random variable. The RM LMM predicting clutch size included the three main effects, with female extra‐pair success included as the fixed effect “Clutch Status (EP or WP only)”; here “EP” denotes that one or more EPOs were present in the nest, and “WP only” denotes that all surviving nestlings were WPOs. Mother's identity was included as a random variable.

The RM LMM predicting nest attendance time included founder sex as a fixed effect in addition to the three main effects, where the fixed effect for female extra‐pair success was “Clutch Status (EP or WP only)”; as for the model predicting clutch size, “EP” denotes that one or more EPOs were present in the nest, and “WP only” denotes that all surviving nestlings were WPOs. Interactions between founder sex and other main effects were considered based on *a priori* expectations of sex differences in nest attendance time (Wilson et al., 2017). Observation day (defined above) was included as a covariate and, since first clutches can be less successful, clutch number was also included as a covariate. Mother's identity was included as a random variable.

The RM LMMs predicting nestling provisioning included nestling sex as a fixed effect in addition to the three main effects, with female extra‐pair success scored as the fixed effect “Nestling Status (EP or WP),” which was assigned to each individual nestling. Nestling age on the day of sampling and clutch number were included as covariates. Nestling identity was included as a random variable.

Three‐way ANOVAs were performed to assess how female extra‐pair success and the two measures of mate attractiveness influenced the total number of offspring that females produced. Numbers of sons and daughters were also analyzed separately since several studies have shown for this species that several environmental and parental conditions influence the relative production of the two sexes (Bradbury & Blakey, 1998; DeKogel, 1997; Foster & Burley, 2007; Kilner, 1998; Martins, 2004). Since the sample size was relatively small (*N* = 25 pairs), interactions were not included in these models. In these analyses, females were dichotomously categorized as having produced 1 or more surviving EPOs (having obtained “extra‐pair success”) or having produced no surviving EPO (not having obtained “extra‐pair success”). Similar two‐way ANOVAs were run to assess variation in production of EPOs by females as a function of social mate attractiveness. Finally, Pearson's tests were used to ask whether the total number of offspring and number of extra‐pair offspring produced by females and their social mates were correlated and Fisher's exact test was performed to determine whether a female's tendency to produce extra‐pair offspring was independent of her social mate's attractiveness.

Analyses were found to meet normality assumptions by visual inspection of quantile–quantile plots and Shapiro–Wilk's tests. Egg mass was log‐transformed in order to meet these assumptions. Marginal means and delta standard errors are reported. Statistical tests were performed in STATA 14 (StataCorp LP, College Station, Texas, USA).

All experiments were carried out in accordance with the Institutional Animal Care and Use Committee at the University of California, Irvine, and were consistent with USA federal guidelines (IACUC protocol 1334‐1998).

## 3 RESULTS

Whether or not a female produced one or more extra‐pair offspring was independent of her social mate's attractiveness (Fisher's exact: male natal diet—*p* = .12; male extra‐pair success—*p* = .39).

### 3.1 Egg mass and clutch effects

**FIGURE 2:**
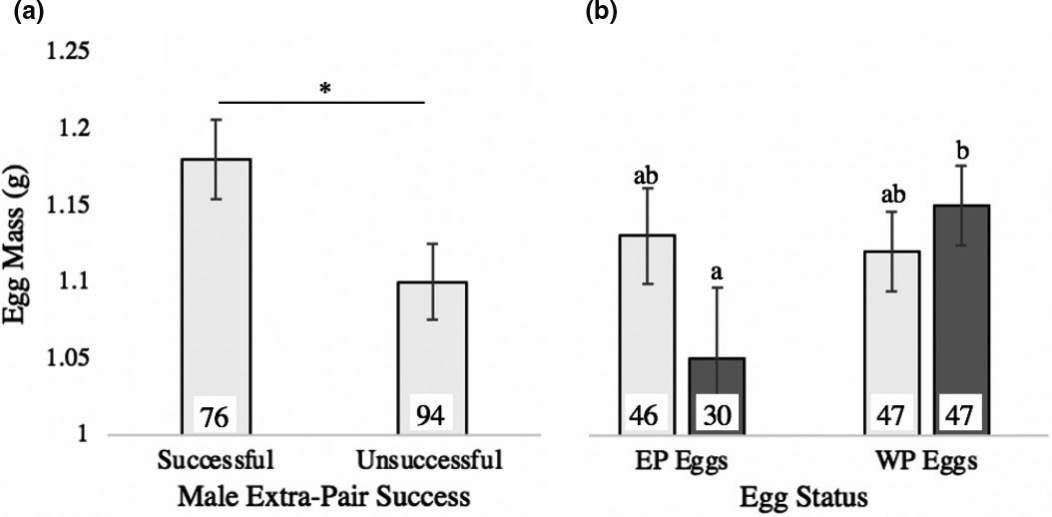
Egg mass patterns for offspring surviving to independence (X̄+/‐ SE). A) Egg mass as a function of extra‐pair success of females’ social mates. B) The effects of male natal diet and egg status (EP or WP) on egg mass. Bar color (1B only) reflects male natal diet (light gray bars—HI‐diet males; dark gray bars—LO‐diet males). Data correspond to Table 2. Lowercase letters indicate location of significant differences among groups based on post hoc analyses (1B—*p* = .009). Sample size (number of eggs) for each group is listed on its corresponding bar

Female investment in egg mass was influenced by lay order, male extra‐pair success, and the interaction between male diet history and EP status of the egg. Egg mass increased significantly with lay order (*p* < .001). Females mated to males that achieved extra‐pair success laid heavier eggs than those mated to males without extra‐pair success (Table 2; Figure 2a). Among females with LO‐diet social mates, EP eggs weighed less than WP eggs (*p* < .001) (Table 2; Figure 2b).

**TABLE 2:** Egg mass and clutch size of offspring surviving to independence

Variable	Test Value	*p*	Model *p*
Egg Mass^a^, ^b^	χ^2^ = 78.56		<.0001
Egg Status (EP or WP)	z = −0.54	.592	
Male Extra‐Pair Success	z = 3.85	<.001	
Male Natal Diet	z = 1.78	.075	
Egg Status (EP or WP) * Male Natal Diet	z = −2.65	.008	
Lay Order	z = 7.09	<.001	
Clutch Size	χ^2^ = 9.04		.0600
Clutch Status (EP or WP only)	z = −1.25	.211	
Male Extra‐Pair Success	z = −0.94	.345	
Male Natal Diet	z = −2.55	.011	
Male Extra‐Pair Success * Male Natal Diet	z = 1.97	.049	

Repeated‐measures linear mixed‐effects models (initial models included: 3 main effects, interactions between male natal diet and other main effects, lay order as a covariate [egg mass analysis only], and female identity as a random effect)—egg mass: N_observations_ = 170, N_mothers_ = 24; clutch size: N_observations_ = 55, N_mothers_ = 24.

^a^ Female identity contributed significantly to the model.

^b^ Log‐transformed.

The model predicting clutch size variation only trended toward significance (LLM: χ^2^ = 9.04; *p* = .06). Among females mated to males without extra‐pair success, those with HI‐diet mates tended to lay larger clutches (X̄ +/‐ SE—unsuccessful/HI diet: 5.17 +/‐ 0.32 eggs, *N* = 13; unsuccessful/LO diet: 4.13 +/‐ 0.26 eggs, *N* = 20) (Table 2). EP eggs were equally likely to be laid in the first, middle, or last third of each clutch (Fisher's exact: *p* = .65).

### 3.2 Incubation‐phase parental effort

**FIGURE 3:**
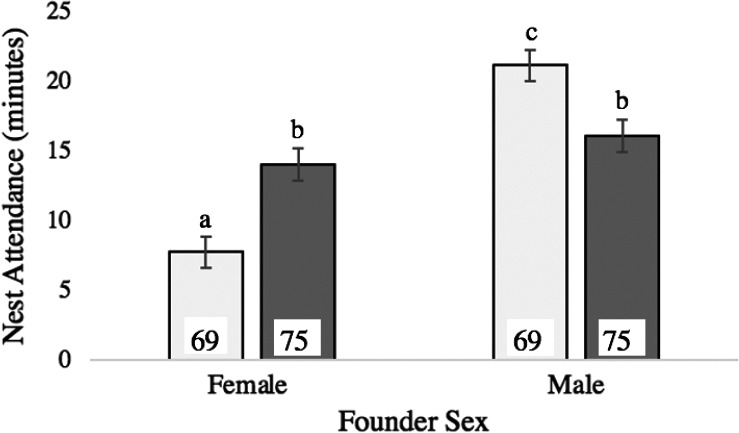
Interaction between founder sex and male extra‐pair success on average nest attendance time (X̄+/‐ SE per 30‐min sample). Light gray bars represent pairs in which males achieved extra‐pair success; dark gray bars represent pairs in which males did not achieve extra‐pair success. Data correspond to Table 3. Lowercase letters indicate location of significant differences among groups based on post hoc analyses (*p* ≤ .028). Sample size (number of observation samples) for each group is listed on its corresponding bar

Nest attendance time was affected by male extra‐pair success and the interaction between male extra‐pair success and breeder sex. Males that achieved extra‐pair success spent the most time attending their nests (*p* < .002), while their mates spent the least (*p* < .001). Males without extra‐pair success spent less time attending their nests than males that achieved extra‐pair success (z = 3.11; *p* = .002) and a similar amount of time as their mates (z = 1.30; *p* = .19) (Table 3; Figure 3).

**TABLE 3:** Nest attendance time (per 30‐min sample) by caregivers during the incubation phase

Variable	Test Value	*p*	Model *p*
Nest attendance time	χ^2^ = 69.66		<.0001
Founder Sex	z = 1.30	.192	
Clutch Status (EP or WP only)	z = 0.42	.673	
Male Extra‐Pair Success	z = −3.90	< .001	
Male Natal Diet	z = −0.46	.645	
Founder Sex*Male Extra‐Pair Success	z = 5.01	< .001	

Repeated‐measures linear mixed‐effects model (initial model included: 3 main effects plus founder sex, interactions between founder sex and other main effects, and female identity as a random effect), N_observations_ = 288, N_clutches_ = 28, N_mothers_ = 18.

While the division of labor varied among pairs as described above, the total amount of time that nests were attended did not vary based on clutch EP status, male extra‐pair offspring production, or male diet history (repeated‐measures LMM: χ^2^ = 0.66; *p* = .88).

### 3.3 Nestling‐phase parental effort

**FIGURE 4:**
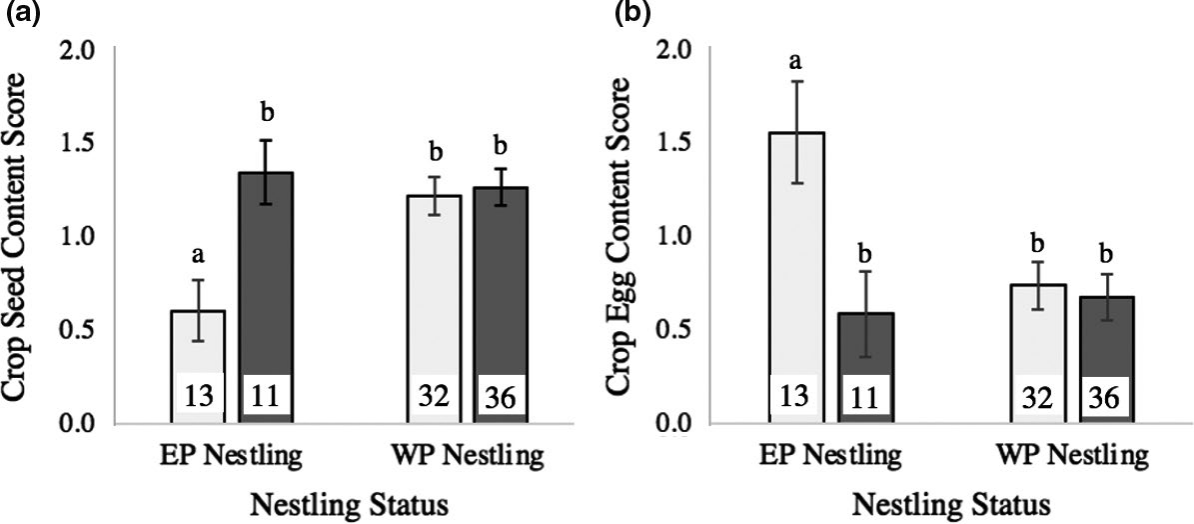
Patterns of nestling provisioning of hen's egg. Crop content score (X +/‐ SE) as a function of nestling status (EPO vs. WPO) and male natal diet. Bar color reflects male natal diet (light gray bars—HI‐diet males; dark gray bars—LO‐diet males). A) Average crop seed content score. B) Average crop egg content score. Data correspond to Table 4. Lowercase letters indicate location of significant differences between groups based on post hoc analyses (*p* ≤ .019). Sample size (number of provisioning samples) for each group is listed on its corresponding bar

Male extra‐pair success and nestling age influenced nestling seed provisioning, and nestling extra‐pair status (EP versus WP) as well as the interaction between nestling extra‐pair status and male natal diet influenced provisioning of both egg and seed. Older nestlings, WP nestlings, and nestlings whose social father produced EPOs elsewhere were provisioned with more seed (crop score X̄ +/‐ SE—produced EPOs: 1.36 +/‐ 0.08; produced WPOs only: 0.95 +/‐ 0.09) (Table 4). By contrast, EP nestlings were provisioned with more egg (crop score X̄ +/‐ SE—EP: 1.12 +/‐ 0.18; WP: 0.71 +/‐ 0.08) and less seed (crop score X̄ +/‐ SE—EP: 1.00 +/‐ 0.12; WP: 1.24 +/‐ 0.06) than WP nestlings. This pattern was driven by the interaction between nestling extra‐pair status and male natal diet (Table 4): EP nestlings with HI‐diet social fathers were provisioned with the highest amounts of egg and the lowest amounts of seed (Figure 4).

**TABLE 4:** Mean crop content scores of nestlings sampled after hen's egg was provided to breeders (seed was available ad libitum)

Variable	Test Value	*p*	Model *p*
Seed Score	χ^2^ = 43.54		<.0001
Nestling Sex	z = 0.36	.718	
Nestling Status (EP or WP)	z = −3.32	.001	
Male Extra‐Pair Success	z = 3.33	.001	
Male Natal Diet	z = 0.32	.752	
Nestling Status (EP or WP)*Male Natal Diet	z = 2.44	.015	
Nestling Age	z = 4.49	<.001	
Hen's Egg Score	χ^2^ = 34.15		<.0001
Nestling Sex	z = −1.63	.103	
Nestling Status (EP or WP)	z = 4.44	<.001	
Male Extra‐Pair Success	z = 0.00	.999	
Male Natal Diet	z = −0.54	.590	
Nestling Status (EP or WP)*Male Natal Diet	z = −2.49	.013	

Repeated‐measures linear mixed‐effects model (initial models included: 3 main effects plus nestling sex, interactions between male natal diet and other main effects, nestling age as a covariate, and nestling identity as a random effect), N_observations_ = 92, N_nestlings_ = 44.

### 3.4 Reproductive success

**FIGURE 5:**
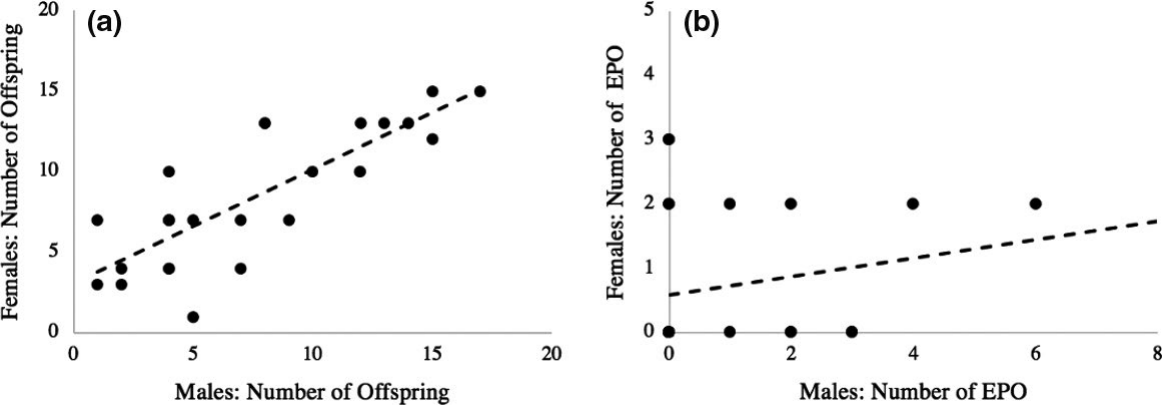
Correlation between reproductive success of social mates. (a) Correlation between the total number of genetic offspring produced by male and female partners (Pearson's R^2^ = 0.811; *p* < .0001). (b) Correlation between the total number of extra‐pair offspring produced by male and female partners (Pearson's R^2^ = 0.207; *p* = .32)

**FIGURE 6:**
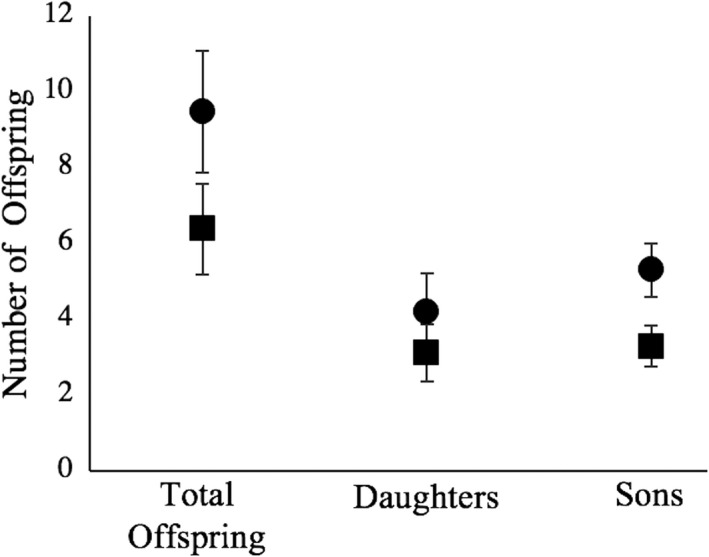
Number of offspring produced by females based on EP success (X̄ +/‐ SE). Circles indicate females that produced one or more surviving EPOs during the study (*N* = 9). Squares indicate females that produced only WPOs (*N* = 16). *p < .05. Data correspond to Table 5

Total reproductive success between social mates was highly correlated (Pearson's correlation: R^2^ = 0.811; *p* < .0001) but EP reproductive success was not (Pearson's correlation: R^2^ = 0.207; *p* = .32) (Figure 5). Analyses revealed predictors of son production, but not daughter production or total offspring production. Females that produced one or more EPOs during the study produced more surviving sons than females that produced only WPOs (Table 5; Figure 6). Males’ natal diet and extra‐pair success did not impact their social mates’ extra‐pair offspring production (*p* > .18; Table 6). Lastly, females did not produce more EPOs of one sex (Fisher's exact *p* = .39).

**TABLE 5:** Number of offspring produced by each female that survived to independence

Variable	Degrees of Freedom	Sum of Squares (Mean Square of the Error)	*F*‐Value	*p*
Total Surviving Offspring	3, 24		1.65	.21
Female Extra‐Pair Success	1	48.32	2.25	.15
Male Extra‐Pair Success	1	9.49	0.44	.51
Male Natal Diet	1	5.59	0.26	.61
Residual	21	450.11 (21.43)		
Male Offspring	3, 24		4.86	.01
Female Extra‐Pair Success	1	20.43	4.85	.039
Male Extra‐Pair Success	1	11.52	2.73	.113
Male Natal Diet	1	2.92	0.69	.415
Residual	21	88.55 (4.22)		
Female Offspring	3, 24		0.31	.82
Female Extra‐Pair Success	1	5.91	0.67	.421
Male Extra‐Pair Success	1	0.10	0.01	.917
Male Natal Diet	1	0.43	0.05	.827
Residual	21	184.06 (8.76)		

3‐way ANOVAs (initial models included 3 main effects only), N_observations_ = 25, N_mothers_ = 25.

**TABLE 6:** Number of extra‐pair offspring produced by each female that survived to independence

Variable	Degrees of Freedom	Sum of Squares (Mean Square of the Error)	*F*‐Value	*p*
Total Surviving EPOs	2, 24		1.76	.196
Male Extra‐Pair Success	1	1.67	0.70	.411
Male Natal Diet	1	3.20	1.34	.259
Residual	22	52.55 (2.39)		
Male EPOs	2, 24		1.32	.300
Male Extra‐Pair Success	1	0.75	1.21	.285
Male Natal Diet	1	0.75	0.21	.654
Residual	22	21.37 (0.97)		
Female EPOs	2, 24		1.83	.183
Male Extra‐Pair Success	1	0.18	0.39	.536
Male Natal Diet	1	0.85	1.87	.186
Residual	22	10.07 (0.46)		

2‐way ANOVAs (initial model included 2 main effects only), N_observations_ = 25, N_mothers_ = 25.

## 4 DISCUSSION

Differential allocation has been demonstrated in both free‐living and captive populations (Burley, 1986; Horvathova et al., 2012). As shown here and elsewhere (e.g., Rutstein et al., 2004; Gilbert et al., 2006; Johnsen et al., 2005), food availability need not be limited to observe patterns of differential allocation, because parental expenditure can be limited by numerous environmental and physiological trade‐offs (e.g., Svensson & Nilsen, 1997; Williams, 2012). Breeders may facultatively increase the number of eggs laid and/or offspring reared per brood when food availability increases, for example, and then become time/energy limited as a result. In short, the availability of ample food in a laboratory environment does not imply that breeders fail to experience costs of parental care (Burley, 1988). In this light, and as discussed in detail below, some patterns described here lend some support to our prediction that females in this experiment would engage in differential allocation: Notably, greater female investment was associated with the presence of EPOs in their broods and with having a high‐quality mate. Additionally, while female success at producing EPOs was not associated with higher total offspring production, females with extra‐pair success had a greater number of surviving sons, which leaves open the possibility that females benefit by producing offspring who will themselves experience greater reproductive success.

It is important for researchers to acknowledge the range of plausible interpretations of their results. Here, we need to keep in mind that variation in mate quality may generate positive assortative mating patterns (Burley, 1986; Holveck & Riebel, 2010) even though various constraints—ecological and informational—on such assortment may lead to differential allocation (Burley, 1986, 2019); given this, it is possible that patterns described here may also relate to assortative mating patterns. Since female mating quality can be difficult to assess, assortative pairing and differential allocation can often not be parsimoniously resolved.

### 4.1 Egg phase

Egg mass increased with laying order but EP eggs were not heavier than WP eggs (Table 2) and, unlike reports on other species (Cordero et al., 1999; Krist et al., 2005; Magrath et al., 2009), in the present study EP eggs were not more likely to be laid near the beginning of a clutch, where mortality is typically lower (Ferree et al., 2010; Magrath et al., 2009). Nonetheless, there was evidence of female differential allocation based on social mate quality during the egg phase: Females produced heavier eggs when mated to males with extra‐pair success (Figure 2a), and females mated to HI‐diet males tended to produce larger clutches (Table 2). Differential allocation toward eggs in response to male quality/attractiveness has been demonstrated previously, both in this species (Gilbert et al., 2006; Arnold et al., 2016; but see Bolund et al., 2009) and other avian species (Cunningham & Russell, 2000; Horvathova et al., 2012). The effects of greater allocation toward offspring sired by attractive males often last into adulthood (Arnold et al., 2016; Cunningham & Russell, 2000; Gilbert et al., 2006), confounding demonstration of possible genetic benefits from sires to offspring.

An alternative, and nonmutually exclusive, interpretation is that pairs formed assortatively based on male attractiveness and female fecundity. Since egg mass is heritable (Christians, 2002; Potti, 1999) and can be influenced by female early‐life environment—with better environments associated with heavier eggs and larger clutches (Griffith & Buchanan, 2010; Monaghan et al., 1996; Potti, 1999)—high‐quality males may have secured mates that were more fecund. Assortative pairing in zebra finches has been reported in both free‐pairing colony experiments (Burley, 1986; Burley & Foster, 2006) and mate choice experiments (Holveck & Riebel, 2010), but it is unclear whether such assortment extends to fecundity, since mixed results are reported for males’ tendency to choose based on female fecundity (Martin & Burley, in press; Wang et al., 2017). Thus, the relative contributions of differential allocation and assortative mating to the egg mass patterns found here remain unclear.

Females paired to LO‐diet males invested less in EP eggs relative to WP eggs (Figure 2b), which is contrary to expectations, since males raised on the LO diet have lower expression of secondary sexual traits (Burley et al., 2018; Wilson et al., 2019), and males successful in obtaining extra‐pair copulations are expected to be of above‐average quality. This finding is, however, potentially consistent with a previous study on this species that reported compensatory investment in eggs in response to social mate quality (Bolund et al., 2009). Specifically, since egg production is costly (Stearns, 1992; Visser & Lessells, 2001; Williams & Miller, 2003), we envision that females with low‐quality social mates may benefit by reducing investment toward individual EP eggs when brood‐mates compete to be fed. Since maternal fitness would likely be reduced if EP offspring—presumed to be of higher genetic quality—were to out‐compete WP offspring for food, a tactic of reduced investment in EP eggs may result in equilibration of brood‐mate competitive ability, while permitting females to produce EP offspring of high genetic quality. The variation in egg mass investment found in this study highlights the potential for genetic and maternal effects to interact in how they drive observed allocation toward EPOs and WPOs. Greater theoretical attention to which aspects of environmental variation are likely to favor production of EPOs (e.g., Eliassen & Kokko, 2008), as well as to the context‐dependent roles of differential allocation and reproductive compensation (Burley, 2019) in production of EPOs and WPOs, would likely advance our understanding of these complex but ecologically relevant patterns.

### 4.2 Incubation phase

Patterns of nest attendance showed differences in how parents divide this shared duty based on male attractiveness but did not suggest occurrence of female differential allocation. Zebra finch pairs exhibit plasticity in incubation behavior (Gilby et al., 2013; Wilson et al., 2017; Zann & Rossetto, 1991), and this plasticity likely reflects differences in individual condition or quality as well as differences in sexual conflict/cooperation (e.g., Gorman & Nager, 2003; Wilson et al., 2017). While avian incubation is typically considered to be female‐led (Burley & Johnson, 2002; Moore & Varricchio, 2016; Tullberg et al., 2002), if a male increases his nest attendance time, a comparable decrease in his social mate's attendance time is expected, since pairs are likely aiming for some optimal total amount of incubation (Jones, 1989; Wilson et al., 2017). Our finding that total nest attendance time did not vary between diet treatments (Table 3) is consistent with this expectation. Although males that sire EPOs are typically predicted to invest less in their social mates’ clutches (due in part to the time and resources required to seek extra‐pair mates—Crouch & Mason‐Gamer, 2018), we found the opposite pattern here (Figure 3). This result may reflect that zebra finches are a gregarious species: They nest in colonies and typically feed in aggregations (McCowan et al., 2015; Zann, 1996). Under these circumstances, male search costs for extra‐pair partners may be low. An important additional consideration is that male quality likely influences the cost of seeking extra‐pair partners: Attractive males should obtain extra‐pair mates with lower effort and may therefore experience little trade‐off between nest attendance and extra‐pair activities (Burley et al., 1994). If so, females mated to such males may then experience greater time to feed early in the clutch cycle, likely contributing to the egg mass pattern observed here (Figure 2a).

### 4.3 Nestling phase

EP nestlings raised by pairs containing attractive males—as measured by early diet quality—received greater provisioning of nutrient‐rich egg. We interpret this pattern as consistent with our prediction of differential allocation of egg resources to offspring of attractive males. To explain this conclusion, we develop several salient points and consider alternative hypotheses. First, we note that the EP nestlings that were fed a greater amount of egg experienced a corresponding decrease in the amount of seed they were fed (Figure 4). This inverse relationship is to be expected given the short sampling interval during which increased provisioning of one food type should result in a decrease in the other. While it is conceivable that the pattern of selective egg provisioning found here could result from WP nestlings having received less egg because their crops were already full of seed, our data do not support this inference, because nestling crops were seldom judged to be full during sampling: Only 22 out of 460 raw samples of nestling crop contents (averaged samples: 5 of 92) were scored higher than about 70% full. Zebra finch parents are more likely to fill the crops of nestlings in late afternoon, when food stores need to last through the night; our sampling was routinely completed before 1,400 hr. One hypothesis to explain the greater egg provisioning of pairs containing HI‐diet males centers on condition effects on risk assessment. When human observers, present to time the 3‐min egg consumption interval prior to provisioning sampling (see Methods), inadvertently caused small disturbances (e.g., by sneezing or dropping a pencil), birds flushed and remained off the floor for 30 s or longer until one or more individuals initiated their return to the egg bowl. Birds in better condition may have assessed the risk to egg foraging to be lower if, for example, they had faster take‐off speeds (Criscuolo et al., 2011; Labocha et al., 2015). However, it is also likely that parents with offspring judged to have higher reproductive value were more willing to take such risks.

Another possible explanation for egg‐provisioning patterns that is important to consider is that LO‐diet males may have had lower tendency to feed egg to offspring as a result of their prior unfamiliarity with egg as a food item. This concern, however, is offset by the finding that males of the two rearing treatments did not differ in their tendency to consume egg in samples collected during the 6‐week interval they spent on the LAB diet prior to the start of the experiment. The palatability of hen's egg to naïve birds has also been demonstrated for wild zebra finches (Burley et al., 1992).

A final possibility important to consider is that egg‐provisioning patterns may have been caused by interference and/or scramble competition, which could influence results, especially if HI‐diet males and their mates were superior competitors. The potential significance of interference competition is offset, however, by the finding of no differences in egg‐eating patterns between HI‐ and LO‐diet males during preliminary trials (see above). More broadly, interference competition at food resources is minimal in our colonies; this pattern likely reflects the natural history of the species, which feeds in flocks on the ground, where birds are vulnerable to predation and where any disruption leads the flock to scatter. In the feeding trials performed here, up to 8 adults at a time crowded onto the rim of the bowl of egg, collected egg in their beaks and quickly flew away; other birds, waiting nearby, then took their turn. The amount of egg was sufficient to ensure that some usually remained after the end of sampling, and trials conducted during protocol development indicated that birds were not excluded from feeding during trials (see Methods); these methods likely minimized effects of scramble competition on results. While we do not contend that competition was entirely absent during feeding trials, the considerations addressed here add weight to our interpretation that the greater relative provisioning to EPOs of HI‐diet males reflects a pattern of differential allocation of high‐quality nutrients to these offspring. While further research to explore questions about foraging risk assessment and the relative roles of the two parents in egg feeding would facilitate interpretation of these results, they are generally consistent with the idea that EPOs have higher reproductive value.

### 4.4 Reproductive success

Contemporary frameworks for the evolution of extra‐pair behavior have highlighted the potential for extra‐pair behavior to be maladaptive for females of pair‐bonding species (Arnqvist & Kirkpatrick, 2005; Forstmeier et al., 2011, 2014; Kempenaers & Schlicht, 2010) but persist in populations due to the benefits of extra‐pair tendencies to males combined with weak or absent sex‐limited expression of such tendencies (Forstmeier et al., 2011). By contrast, our results suggest that females may obtain a fitness benefit by producing more sons, a result that complements a previous report of female zebra finches investing more reproductive resources in sons sired by EP males (Tschirren et al., 2012). In theory, such benefits could arise through greater caregiving toward EPOs and their clutch mates (as seen here in selective egg feeding patterns) which may enhance survival as well as mating quality of offspring in EPO clutches. Collectively, results point to the importance of remaining open‐minded about the possibility that females may obtain direct fitness benefits from extra‐pair activities through changes in parental investment as well as the more frequently invoked possibility of indirect fitness benefits (Akçay & Roughgarden, 2007; Birkhead & Pizarri, 2002; Iwasa & Pomiankowski, 1991; Jennions & Petrie, 2000; Neff & Pitcher, 2005; Tschirren et al., 2012). Another possibility that merits investigation is that those females predisposed to producing more sons on the basis of genetics and/or condition are more likely to accept extra‐pair copulations. Such females might stand to gain more from EPOs, since sons produced with an EP male are expected to have high reproductive value.

## 5 CONCLUSION

Results of this paper indicate that greater attention to the effects of extra‐pair mating on differential allocation of resources to offspring will be a productive avenue for both experimental and theoretical approaches to questions about if and how females may benefit from producing extra‐pair offspring. While findings here are based on a moderate sample size (55 clutches and 52 reproductively active birds), they do present an exciting new direction for behavioral ecology by showing that investment in offspring varies throughout the reproductive phases in response to the extra‐pair success of both nest attendants.

## CONFLICT OF INTEREST

KMW and NTB declare no conflicts of interests.

## AUTHOR CONTRIBUTIONS

**Kerianne Murphy Wilson:** Data curation (equal); Formal analysis (lead); Investigation (equal); Project administration (equal); Writing‐original draft (lead); Writing‐review & editing (equal). **Nancy Tyler Burley:** Conceptualization (lead); Data curation (equal); Formal analysis (supporting); Funding acquisition (supporting); Investigation (equal); Methodology (lead); Project administration (equal); Writing‐original draft (supporting); Writing‐review & editing (equal).

## Data Availability

Data were archived with Dryad: https://doi.org/10.5061/dryad.fqz612js7.
